# Modeling the effect of *PTPN22 *in rheumatoid arthritis

**DOI:** 10.1186/1753-6561-1-s1-s37

**Published:** 2007-12-18

**Authors:** Mathieu Bourgey, Hervé Perdry, Françoise Clerget-Darpoux

**Affiliations:** 1 INSERM U535, BP 1000, Villejuif, 94817 France and Université Paris-Sud, IFR 69, UMR-S535 Villejuif, 94817, France

## Abstract

In order to model the effect of *PTPN22 *on rheumatoid arthritis (RA), we determined the combination of single-nucleotide-polymorphisms (SNPs) showing the strongest association with RA. Three SNPs (rs2476601-rs12730735-rs11102685) were selected for which we estimated the genotypic relative risks (GRRs) of the corresponding genotypes. On the basis of these GRRs we defined four at-risk genotypic classes. Relative to the class of reference risk, individuals had a risk approximately multiplied by two, three, or four. This classification was confirmed by the excess of identity-by-descent (IBD) sharing (IBD = 2) for the sibs of an index in the high-risk class and by excess of non-IBD sharing (IBD = 0) when the index belonged to the low-risk class. The observed data could not be explained by the role of a single variant but were compatible either with a joint effect of the three typed SNPs of *PTPN22 *on RA or with the role of two untyped variants.

## Background

The single-nucleotide polymorphism (SNP) R620W, also denoted rs2476601, is located within the hematopoietic-specific protein tyrosine phosphatase gene, *PTPN22*. This SNP (C/T) codes for an amino-acid change and the frequency of its minor allele T has been recently and repeatedly shown to be increased in patients with rheumatoid arthritis (RA) [1]. The allele T confers 1.7- to 1.9-fold increased risk to heterozygote and higher risks to homozygote carriers [2] compared to the non-carrier individuals. This variant is also well known to be associated with several other autoimmune diseases [3], such as systemic lupus erythematosus and type 1 diabetes. Recently, Carlton et al. [2] studied the *PTPN22 *genetic variations in the North American Rheumatoid Arthritis Consortium (NARAC) data. Using the information on several SNPs typed in *PTPN22*, they compared the haplotype distributions in NARAC patients and controls. They demonstrated that SNP R620W does not fully explain the association between *PTPN22 *and RA and suggested the effect of at least one additional variant in the *PTPN22 *gene.

We propose here to reanalyze the NARAC data using both association and linkage information for modeling the role of *PTPN22 *in RA.

## Methods

### Data

We selected from the NARAC data the 511 families with affected sib pairs typed for 14 SNPs of *PTPN22*, and 1404 unrelated controls also typed for all these SNPs. For each affected sib pair we considered the proband as an index RA patient. The R620W SNP is one of the 14 SNPs in *PTPN22*. It is located at the ninth position, so it will be subsequently denoted as SNP 9. A preliminary study of linkage disequilibrium (LD) among the 14 SNPs was examined in the 1404 controls. The LD analysis lead us to exclude 3 SNPs (SNP 2, SNP 12, SNP 13), which are in complete LD with one (or more) other SNP(s).

### Selection of associated SNPs

The combination test [4] was used on the 11 remaining SNPs to select the subset of SNPs showing a significant difference in the genotypic distribution between RA index patients and controls. Its principle consists in testing all possible combinations of SNPs within a gene. Here, there are (2^11^-1) possible combinations. Such a systematic testing of all SNPs and all SNP combinations raises the problem of multiple and non-independent tests. This problem is generally solved by the implementation of a permutation procedure that allows estimation of corrected *p*-values. Here, associated combinations are very significant and the number of permutations necessary to discriminate them would be extremely high and almost unreachable. Nevertheless, the chi-square values of the genotypic association test are so high that even the conservative Bonferroni correction can be used. We selected the most associated and parsimonious subset of SNPs by nested chi-square tests (NCST) in a forward procedure. The NCST compares the strength of association between nested significant subsets.

### Genotypic relative-risk estimation

For the selected subset of SNPs, we used the marker association segregation chi-square (MASC) method [5] to compute the genotypic relative risk (GRR) of each genotype. The genotype distributions of index and controls was conditional on the fact that the index has an affected sib.

### Stratified sib pair IBD estimation

Conditional on each marker genotype of the index cases, the number of parental alleles identical by descent (IBD) shared by the index case and one affected sib were estimated on *PTPN22 *with the MERLIN software [6]. MERLIN is able to take into account LD between SNPs during the IBD computation. So the estimated IBD distributions are computed on the overall set of SNPs even if they are in LD. The fit of a model to the IBD distributions stratified on index marker genotypes [7] may then be tested by the MASC method.

### Modeling PTPN22 effect

We applied the MASC method [5] to find the most parsimonious model explaining the overall observations, i.e., the genotype and the stratified sib-pair IBD distributions. To do this, MASC requires the haplotype frequencies in the general population, which were estimated on the unrelated controls by the MERLIN software.

The MASC method computes the expected genotype marker distribution and the expected sib-pair IBD distributions stratified on marker genotypes for a given genetic model. Here, the computation of the genotypic distribution is conditioned on the fact that index cases have an affected sib. The global expected likelihood of the genetic model given the observed data is then computed as the product of the likelihoods of each expected distribution, and is maximized on the model parameters. The fit of the model to the observed data is tested by a likelihood ratio test (LRT) between global expected likelihood and the likelihood of the saturated model.

## Results

### Selection of associated SNPs

Many subsets of SNPs show significant associations. Table 1 presents a selection of the most associated combinations of one, two, and three SNPs. When considering only the effect of a single SNP, the only significant associated one after correction for multiple testing is SNP 9. The combination of SNPs 9–10 is the one which, among the combination of two SNPs, best improves the association shown by the SNP 9 alone (*p *= 0.017). The subset SNPs 9-10-11 (rs2476601-rs12730735-rs11102685) is the only one that improves significantly the association shown by the SNPs 9–10 (*p *= 0.038). Adding another SNP to this subset does not significantly improve the association. Consequently, all the subsequent analyses have been done considering SNPs 9-10-11 and their ten corresponding genotypes.

**Table 1:** Most associated subsets of one, two and three SNPs

Subset	χ^2 ^df	χ^2 ^value	Corrected *p*-value	Compared subsets	NCST df	NCST value	NCST *p*-value
9	2	47.6	9.5×10^-8^	-	-	-	-
4_9	4	54.0	11.0×10^-8^	4_9 vs. 9	2	6.4	0.041
9_10	7	61.3	17.2×10^-8^	9_10 vs. 9	5	13.7	0.017
9_11	5	57.3	9.2×10^-8^	9_11 vs. 9	3	9.7	0.021
9_10_11	12	73.0	17.8×10^-8^	9_10_11 vs. 9_10	5	11.7	0.038

### GRR estimation

Table 2 displays the genotypes and the corresponding GRRs for SNP 9 taken alone (columns 1 and 2) and for the set of the three SNPs 9-10-11 (columns 3 and 4). The GRRs vary from 1 to 2.7 when considering only SNP 9, whereas the variation ranges from 1 to 4.7 when the information on the three SNPs is taken into account. Interestingly, the CC genotype of the SNP 9 can be subdivided in several genotypes when taking into account the genotypes for SNPs 10 and 11 (rows 1 to 6) with GRRs ranging from 1 (CC-GG-AA) to 3.6 (CC-AA-GG). This observation demonstrates the importance of using the additional information on SNPs 10–11.

**Table 2:** GRR estimates

SNP 9	GRR	SNP 9-10-11	GRR
CC	1	CC-AA-AA	1.60
		CC-AA-AG	1.76
		CC-AA-GG	3.60
		CC-AG-AA	1.73
		CC-*G-AG^a^	2.35
		CC-GG-AA	1
			
CT	1.66	CT-AA-AA	2.88
		CT-AA-AG	3.11
		CT-AG-AA	2.61
			
TT	2.7	TT-A*-AA^a^	4.68

^a ^*, either the A or the G alleles of SNP 10.

### Sib pair IBD estimation

The proportion of RA sibs sharing 0, 1, or 2 parental alleles for PTPN22 is 0.26 (181 pairs), 0.51 (362 pairs), and 0.23 (167 pairs), respectively, and does not differ from the IBD distribution 0.25; 0.5; 0.25 expected under no linkage. However, if our GRRs correctly reflect the differential risk of RA, we expect to see differences in the IBD vectors stratified on the genotypes of the subset of SNPs 9-10-11 [7]. To avoid cells with small numbers of individuals we pooled sib pairs with the index genotypes (SNP 9-10-11) that have similar risk. We thus defined four arbitrary at risk genotypic classes: the low risk class (L; GRR = 1; 19 pairs), the intermediate risk class 1 (I1; 1 < GRR ≤ 2; 295 pairs), the intermediate risk class 2 (I2; 2 < GRR ≤ 3; 157 pairs), and the high risk class (H; GRR > 3; 34 pairs).

Table 3 shows that the proportion of IBD = 0 decreases from 0.47 to 0.09 according to the fact that the index belongs to class L or class H and conversely, the proportion of IBD = 2 increases from 0.11 to 0.26. These stratified IBD distributions are consistent with the risk genotypic classes. In contrast, the IBD sharing distributions stratified only on SNP 9 genotypes are not consistent with the GRR estimates on this SNP (Table 4).

**Table 3:** IBD distribution stratified on the index classes for the four risk classes number of individuals (in parentheses)

Risk class	IBD = 0	IBD = 1	IBD = 2
L	0.47 (9)	0.42 (8)	0.11 (2)
I1	0.29 (85)	0.49 (146)	0.22 (64)
I2	0.26 (41)	0.50 (78)	0.24 (38)
H	0.09 (3)	0.65 (22)	0.26 (9)

**Table 4:** IBD distribution for the three genotypes of the SNP 9 number of individuals (in parentheses)

Genotype of index	IBD = 0	IBD = 1	IBD = 2
CC	0.27 (96)	0.49 (177)	0.24 (85)
CT	0.25 (35)	0.53 (75)	0.22 (31)
TT	0.14 (2)	0.65 (10)	0.21 (3)

^a^IBD distributions stratified on the index genotypes are given in proportions and in effectives.

### Modeling PTPN22 effect

We apply the MASC method in using the genotype distribution only on the SNP 9 and the IBD stratified on the SNP 9 genotypes. In that case, the single and causal effect of the SNP 9 is not rejected (*p *= 0.29). Then, we model the effect of *PTPN22 *using the four genotypic groups of risk defined on the genotypes of the combination of the SNPs 9-10-11 and the IBD information stratified on them. In this case, we reject the direct effect of SNP 9 (*p *= 0.005). We also reject the effect of a single untyped SNP (*p *= 0.04). However, we do not reject the interactive effect of the 3 SNPs (*p *= 0.53) or the interactive effect of two untyped SNPs.

## Discussion

The involvement of *PTPN22 *and *HLA *in RA susceptibility is no longer disputed. However, as shown by Carlton et al. and confirmed in this study, the role of PTPN22 cannot be explained only by the R620W SNP.

A correct modeling of *PTPN22 *is important and shows that the genotypic risk varies much more (1 to 4.7) than reported in the literature (1 to 2.7) [4]. In this study we proposed, for the first time, a model for the effect of *PTPN22*, taking into account both association and linkage information.

Another method, called LAMP [8] was recently proposed for joint modeling of linkage and association [8]. The linkage information used by the LAMP method is the global IBD sharing of affected sib pairs. However, it is very important to note that the power of model discrimination strongly depends on the association and linkage information that is used. As shown here, the information on SNP 9 alone and on the global IBD is very poor as compared with that of the three SNPs 9-10-11 and to the stratified IBD distributions on the four at-risk genotype groups.

In conclusion, we applied a four-step strategy to model the effect of a candidate gene covered by several SNPs: 1) to select the most associated set of SNPs; 2) to group the corresponding genotypes according their GRRs; 3) to stratify IBD sharing information on the at-risk genotype groups; 4) to model the effect of the candidate gene while taking into account both linkage and association information.

This strategy allowed better modeling of the effect of *PTPN22 *in RA susceptibility. Recently, du Montcel et al. [9] refined the modeling of *HLA *in RA susceptibility. A next step will be to use simultaneously the *PTPN22 *and *HLA *information to evaluate their joint effects while taking into account important covariables such as age and gender.

## Competing interests

The author(s) declare that they have no competing interests.

## References

[B1] Begovich AB, Carlton VE, Honigberg LA, Schrodi SJ, Chokkalingam AP, Alexander HC, Ardlie KG, Huang Q, Smith AM, Spoerke JM, Conn MT, Chang M, Chang SY, Saiki RK, Catanese JJ, Leong DU, Garcia VE, McAllister LB, Jeffery DA, Lee AT, Batliwalla F, Remmers E, Criswell LA, Seldin MF, Kastner DL, Amos CI, Sninsky JJ, Gregersen PK (2004). A missense single-nucleotide polymorphism in a gene encoding a protein tyrosine phosphatase (*PTPN22*) is associated with rheumatoid arthritis. Am J Hum Genet.

[B2] Carlton VE, Hu X, Chokkalingam AP, Schrodi SJ, Brandon R, Alexander HC, Chang M, Catanese JJ, Leong DU, Ardlie KG, Kastner DL, Seldin MF, Criswell LA, Gregersen PK, Beasley E, Thomson G, Amos CI, Begovich AB (2005). *PTPN22 *genetic variation: evidence for multiple variants associated with rheumatoid arthritis. Am J Hum Genet.

[B3] Criswell LA, Pfeiffer KA, Lum RF, Gonzales B, Novitzke J, Kern M, Moser KL, Begovich AB, Carlton VE, Li W, Lee AT, Ortmann W, Behrens TW, Gregersen PK (2005). Analysis of families in the multiple autoimmune disease genetics consortium (MADGC) collection: the *PTPN22 *620W allele associates with multiple autoimmune phenotypes. Am J Hum Genet.

[B4] Jannot AS, Essioux L, Reese MG, Clerget-Darpoux F (2003). Improved use of SNP information to detect the role of genes. Genet Epidemiol.

[B5] Clerget-Darpoux F, Babron MC, Prum B, Lathrop GM, Deschamps I, Hors J (1988). A new method to test genetic models in HLA associated diseases: the MASC method. Ann Hum Genet.

[B6] Abecasis GR, Cherny SS, Cookson WO, Cardon LR (2002). Merlin – rapid analysis of dense genetic maps using sparse gene flow trees. Nat Genet.

[B7] Clerget-Darpoux F, Babron MC, Bickeboller H (1995). Comparing the power of linkage detection by the transmission disequilibrium test and the identity-by-descent test. Genet Epidemiol.

[B8] Li M, Boehnke M, Abecasis GR (2005). Joint modeling of linkage and association: identifying SNPs responsible for a linkage signal. Am J Hum Genet.

[B9] du Montcel ST, Michou L, Petit-Teixeira E, Osorio J, Lemaire I, Lasbleiz S, Pierlot C, Quillet P, Bardin T, Prum B, Cornelis F, Clerget-Darpoux F (2005). New classification of HLA-DRB1 alleles supports the shared epitope hypothesis of rheumatoid arthritis susceptibility. Arthritis Rheum.

